# Supplementary material for: Effective binning of metagenomic contigs using contrastive multi-view representation learning
Source: Nat Commun. 2024 Jan 17;15:585. doi: 10.1038/s41467-023-44290-z (PMC10794208; doi:10.1038/s41467-023-44290-z)
Supplement: Supplementary file 1 — Supplementary Information [file 41467_2023_44290_MOESM1_ESM.pdf]

Supplementary Materials for "Effective binning of metagenomic contigs using contrastive multi-view representation learning"

Ziye Wang[1], Ronghui You[1], Haitao Han[1], Wei Liu[1], Fengzhu Sun[2] and Shanfeng Zhu[1,3,4,5,6*]

[1]Institute of Science and Technology for Brain-Inspired Intelligence and MOE Frontiers Center for Brain Science, Fudan University, Shanghai, China.
[2]Department of Quantitative and Computational Biology, University of Southern California, Los Angeles, CA, USA.
[3]Shanghai Qi Zhi Institute, Shanghai, China.
[4]Key Laboratory of Computational Neuroscience and Brain-Inspired Intelligence (Fudan University), Ministry of Education, Shanghai, China.
[5]Shanghai Key Lab of Intelligent Information Processing and Shanghai Institute of Artificial Intelligence Algorithm, Fudan University, Shanghai, China.
[6]Zhangjiang Fudan International Innovation Center, Shanghai, China.

*Corresponding author(s). E-mail(s): zhusf@fudan.edu.cn;

1 Supplementary Figures and Tables

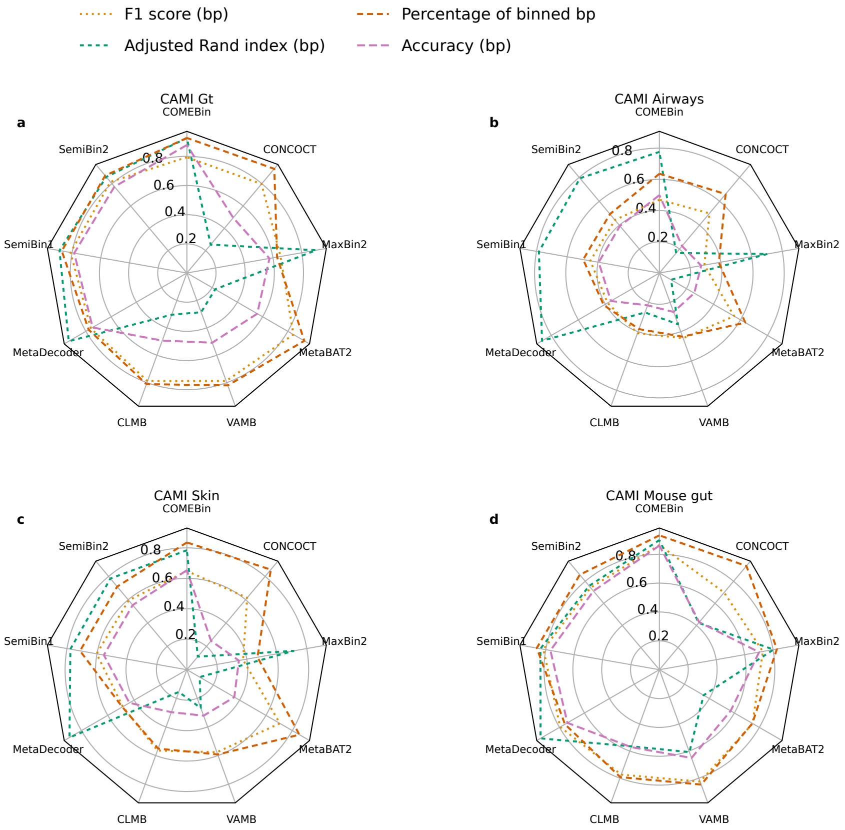

Fig. S1 Comparison of binning methods on four simulated datasets based on the F1-score (bp), Adjusted Rand Index(bp), percentage of binned bp, and accuracy (bp) metrics. **a,** CAMI Gt dataset; **b,** CAMI Airways dataset; **c,** CAMI Skin dataset; and **d,** CAMI Mouse gut dataset.

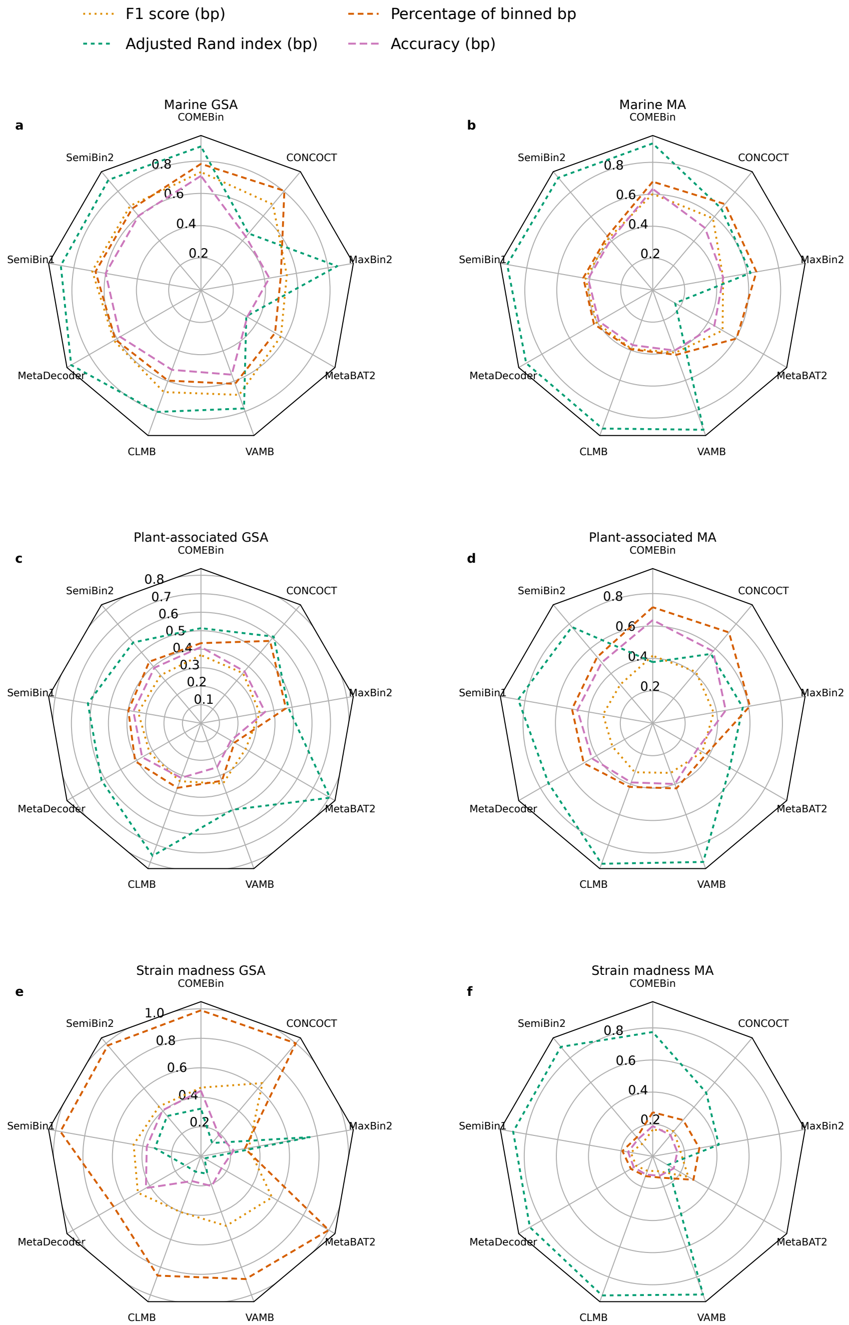

Fig. S2 Comparison of binning methods on CAMI II datasets based on the F1-score (bp), Adjusted Rand Index (bp), percentage of binned bp, and accuracy (bp) metrics. **a,** Marine GSA dataset; **b,** Marine MA dataset; **c,** Plant-associated GSA dataset; **d,** Plant-associated MA dataset; **e,** Strain madness GSA dataset; and **f,** Strain madness MA dataset.

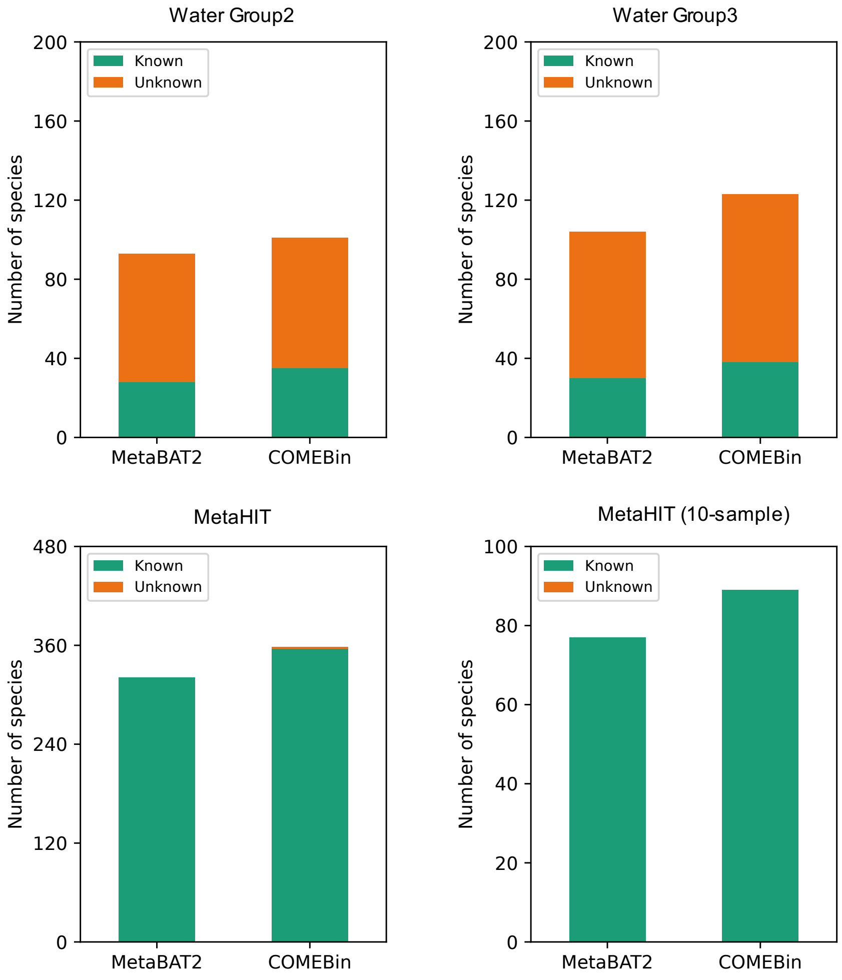

Fig. S3 COMEBin recovers more known and unknown bins with >50% completeness and <5% contamination on the species level. The "known" genomes refer to bins that can be annotated at the species level using GTDB-Tk, and "unknown" otherwise.

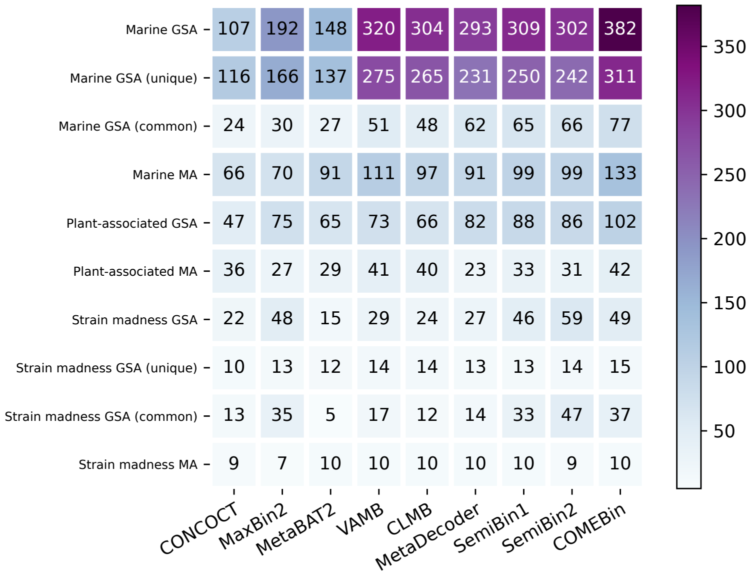

Fig. S4 Comparison of the number of bins with F1-score>0.9 recovered by the binning algorithm. "Unique" denotes the unique strains (genomes with an average nucleotide identity (ANI) of less than 95% to any other genome) introduced in the benchmark paper [1], and "common" otherwise.

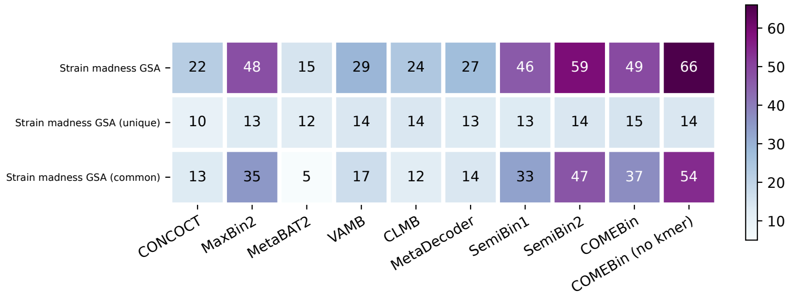

Fig. S5 Comparison of the number of bins with F1-score>0.9 recovered by the binning algorithm on the Strain madness GSA dataset. "Unique" denotes the unique strains (genomes with an average nucleotide identity (ANI) of less than 95% to any other genome) introduced in the benchmark paper [1], and "common" otherwise.

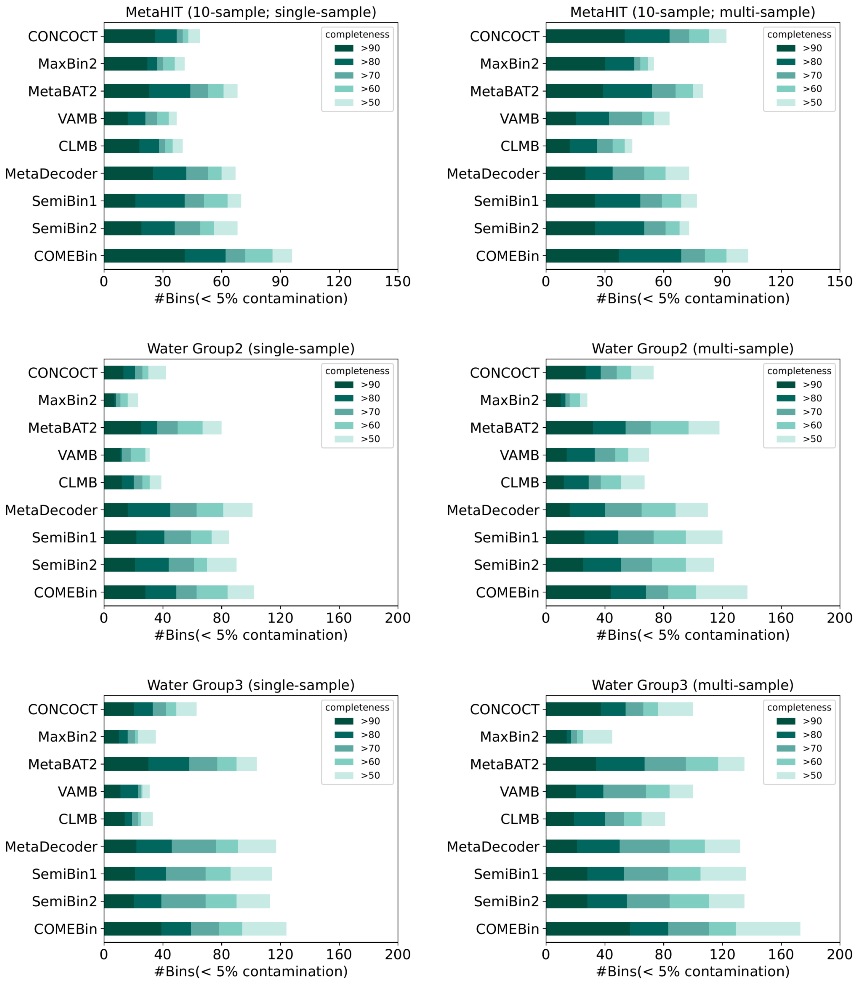

Fig. S6 COMEBin outperforms other binners in real datasets in single- and multi-sample binning.

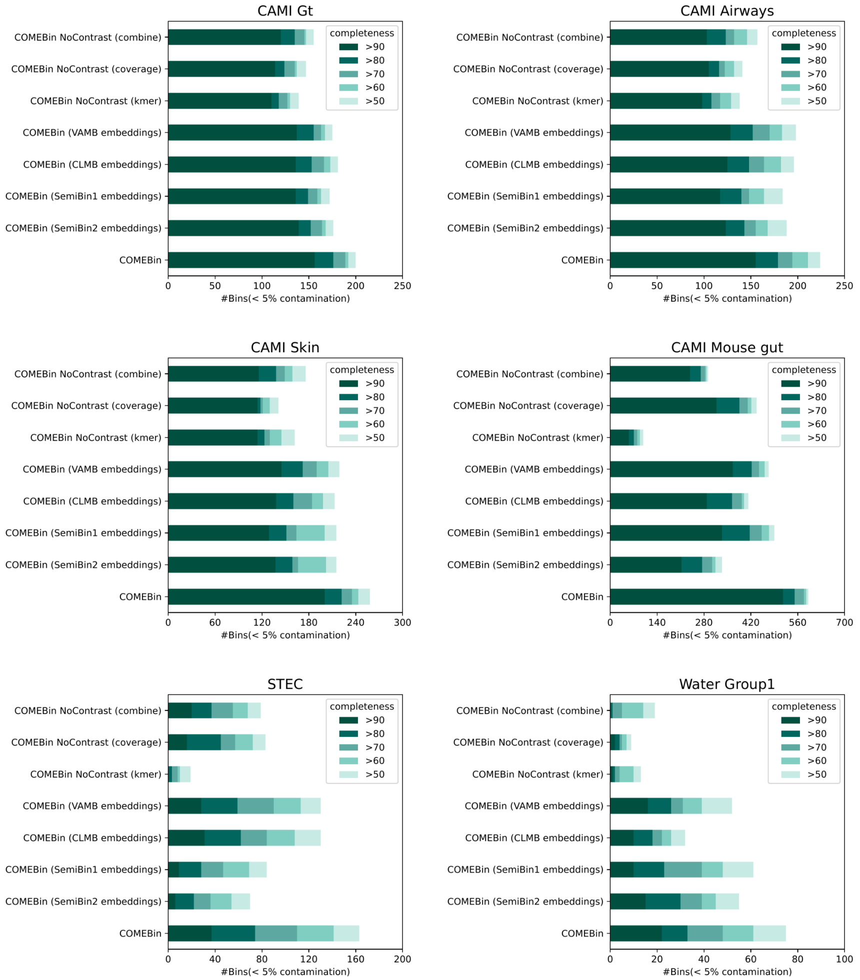

Fig. S7 Comparison of variants of COMEBin. We conducted experiments with different variants of COMEBin by replacing COMEBin embeddings with those from other methods. Subsequently, we applied the same clustering approach used in COMEBin for binning.

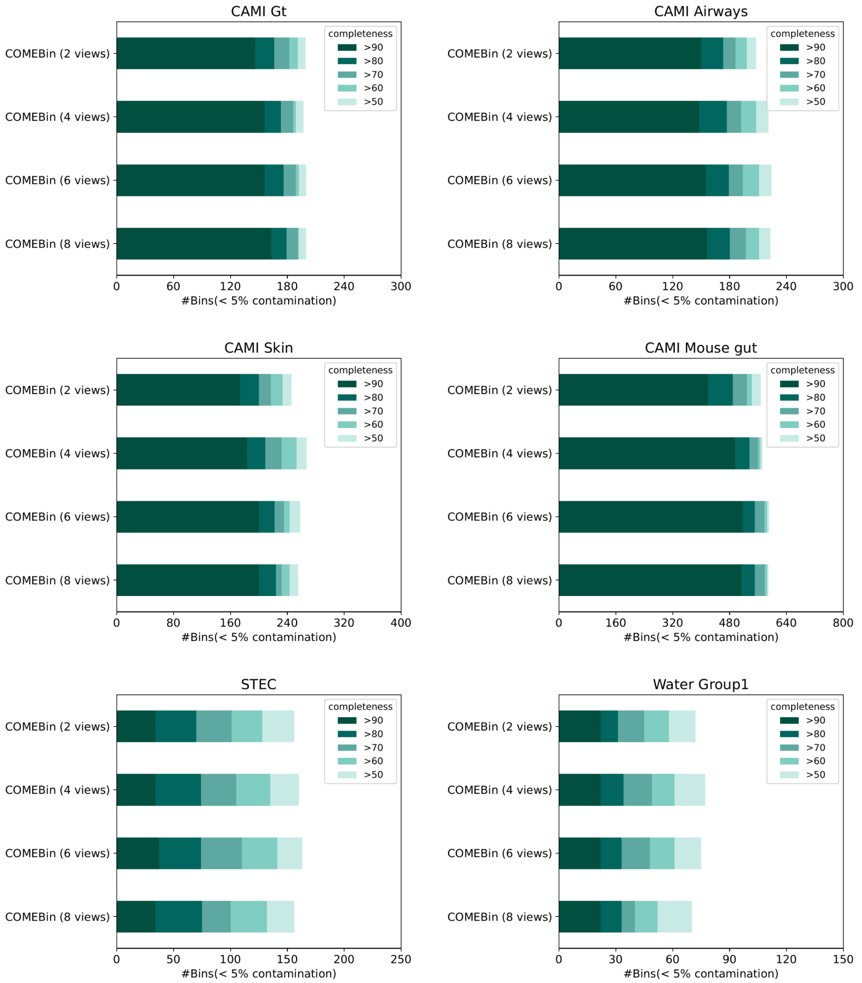

Fig. S8 Comparison of COMEBin with different numbers of views. The number of views indicates the number of sequence fragments extracted from each original contig for augmentation. A view count of six implies that we randomly sampled five sequence segments for augmentation from each original contig, resulting in six views, including the original (original contig). The default setting for COMEBin is six views.

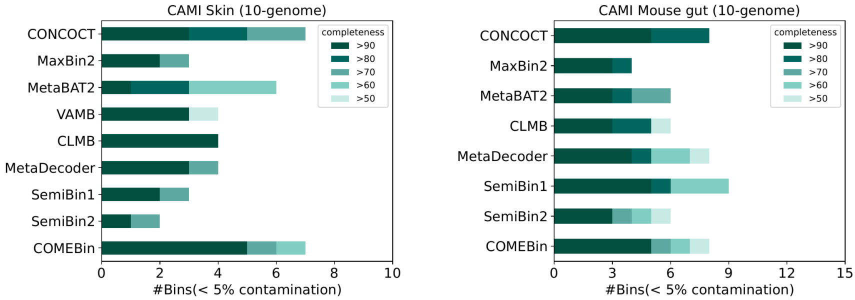

Fig. S9 Comparison of binning methods on two low-complexity datasets. Note that default settings of VAMB are not applicable to the CAMI mouse gut (10-genome) dataset, as the dataset contains fewer than 4096 contigs.

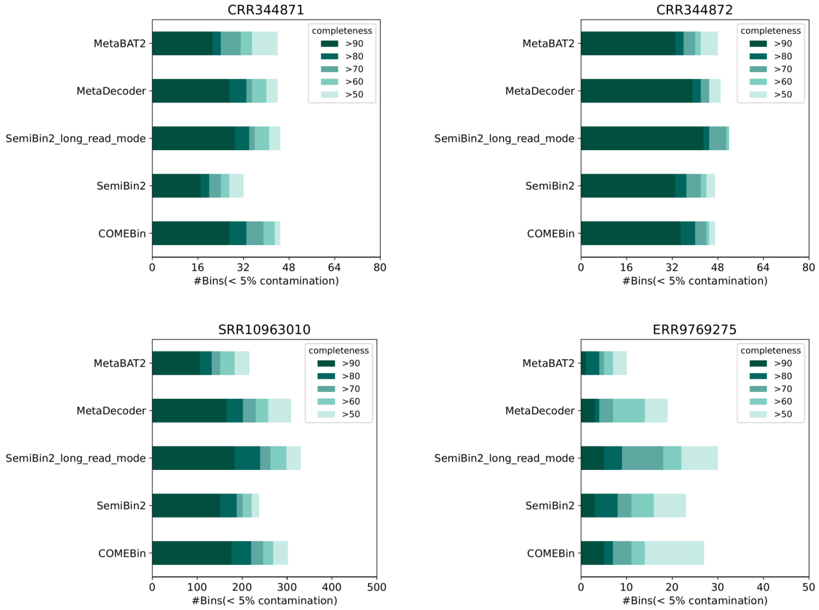

Fig. S10 Comparison of binning methods on long-read sequencing datasets.

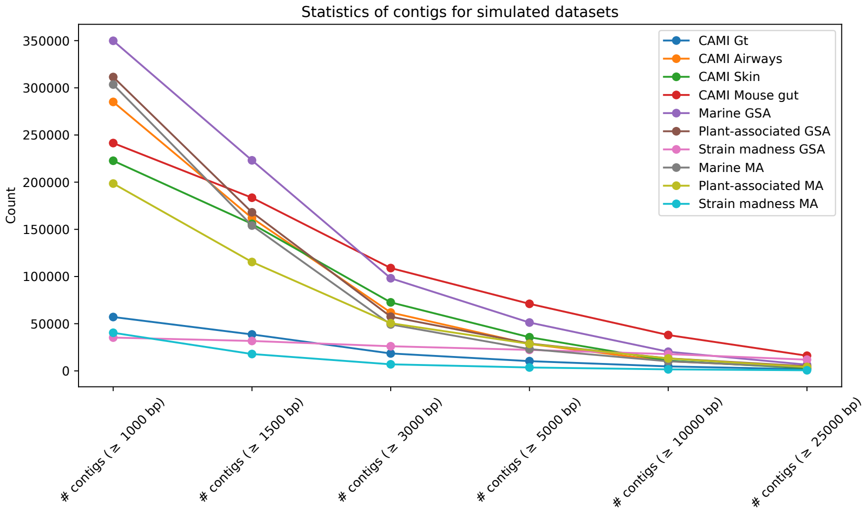

Fig. S11 Sequence length distribution for the simulated datasets.

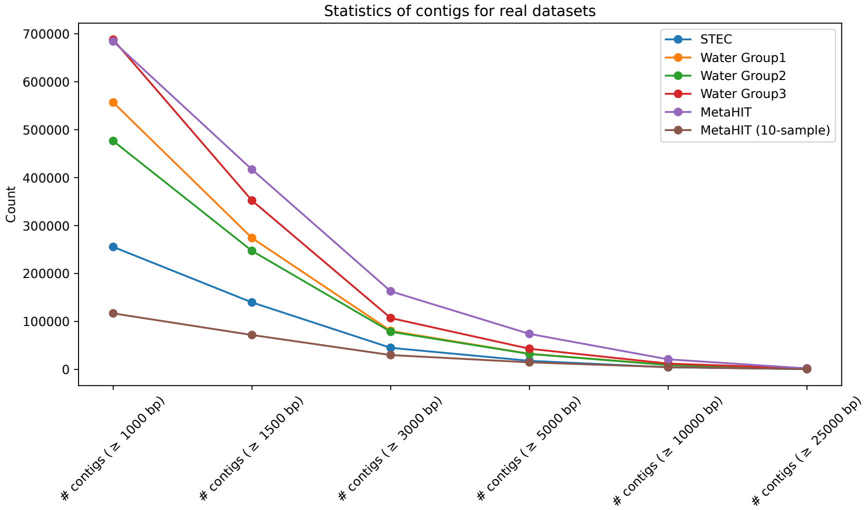

Fig. S12 Sequence length distribution for the real datasets.

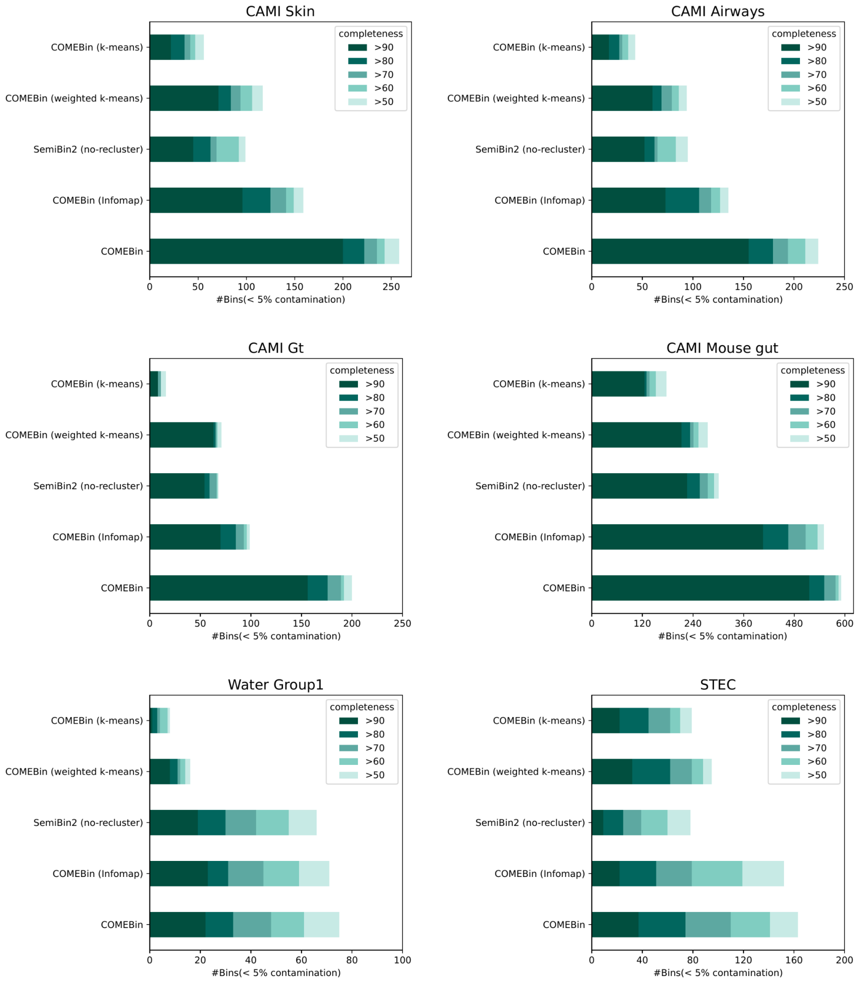

Fig. S13 Comparison of variants of COMEBin using different clustering methods.

Table S1 Running time and memory usage for different datasets and binning modes

Method	BATS (single-sample, average)	BATS (multi-sample, average)	CAMI Gt	STEC	BATS (single-sample, average)	BATS (multi-sample, average)	CAMI Gt	STEC
	Time (min)	Time (min)	Time (min)	Time (min)	Memory (GB)	Memory (GB)	Memory (GB)	Memory (GB)
COMEBin(GPU)	33.07 (±0.53)	33.24 (±0.67)	87.99 (±0.24)	246.33 (±6.99)	5.00 (±0.01)	5.02 (±0.01)	10.77 (±0.09)	10.10 (±0.00)
COMEBin(CPU)	295.52 (±9.04)	288.38 (±9.07)	328.06 (±18.23)	2614.54 (±87.43)	6.30 (±0.01)	6.34 (±0.02)	10.72 (±0.04)	10.09 (±0.00)
SemiBin2(GPU)	24.60 (±12.32)	11.76 (±0.27)	75.94 (±0.43)	64.31 (±1.15)	3.65 (±0.01)	3.64 (±0.01)	5.97 (±0.02)	6.30 (±0.03)
SemiBin2(CPU)	33.84 (±11.93)	18.64 (±0.19)	105.96 (±0.56)	97.09 (±2.50)	1.50 (±0.01)	1.50 (±0.01)	3.80 (±0.02)	4.15 (±0.04)
VAMB(GPU)	6.99 (±0.07)	14.26* (±0.03)	13.19 (±0.08)	34.36 (±0.21)	2.68 (±0.0)	2.79* (±0.01)	3.00 (±0.01)	3.23 (±0.01)
VAMB(CPU)	5.03 (±0.05)	15.36* (±0.08)	13.19 (±0.13)	218.04 (±0.37)	0.55 (±0.0)	0.78* (±0.01)	0.77 (±0.02)	1.39 (±0.02)
# Contigs	15214 to 56346	15214 to 56346	57088	255484	15214 to 56346	15214 to 56346	57088	255484
# Samples used for coverage information	1	10	10	53	1	10	10	53

The results annotated with an asterisk (*) represent the total runtimes or memory usage across all ten samples in VAMB's multi-sample mode. "BATS (average)" represents the average running time or memory usage across the ten BATS samples. We ran each tool on each dataset three times and reported the mean scores and the respective standard deviations.

Table S2 Sample information of the MetaHIT (10-sample) and Bermuda-Atlantic Time-series Study (BATS) samples.

Dataset	Run accessions
MetaHIT (10-sample)	ERR011101
	ERR011113
	ERR011120
	ERR011132
	ERR011152
	ERR011172
	ERR011223
	ERR011284
	ERR011295
	ERR011325
Bermuda-Atlantic Time-series Study (BATS) samples	SRR5720333
	SRR5720340
	SRR5720336
	SRR5720342
	SRR5720343
	SRR5720233
	SRR5720280
	SRR5720278
	SRR5720283
	SRR5720282

The sequencing reads of the BATS samples were downloaded from the NCBI Sequence Read Archive database with accession number PRJNA385855, and the corresponding assemblies were downloaded from the European Nucleotide Archive (ENA) with accession number PRJEB45951.

Table S3 Hyper-parameters used by the network module in the experiments.

Module	#hidden layer	#hidden units	Input size	Output size	Use batch normalization	Activation function
Coverage network	3	2048	#sequencing samples	128	✓	LeakyReLU
Combine network	3	2048	264	128	✓	LeakyReLU

"#hidden layers" denotes the number of hidden layer; "#hidden units" denotes the number of hidden units; "#sequencing samples" denotes the number of sequencing samples.

Table S4 Simulated datasets used in the experiments.

Dataset	The number of samples	The number of contigs (> 1000bp)	N50	Read length (bp)	Sequencing platform (simulated)
CAMI Gt	10	57088	2649095	150	Illumina HiSeq
CAMI Airways	10	285047	13323	150	Illumina HiSeq
CAMI Skin	10	222697	18389	150	Illumina HiSeq
CAMI Mouse gut	64	241451	56781	150	Illumina HiSeq
Marine GSA	10	349831	29227	150	Illumina HiSeq
Marine MA	10	303451	4591	150	Illumina HiSeq
Plant-associated GSA	21	311446	26031	150	Illumina HiSeq
Plant-associated MA	21	198533	8080	150	Illumina HiSeq
Strain-madness GSA	100	35272	137588	150	Illumina HiSeq
Strain-madness MA	100	40385	7456	150	Illumina HiSeq

Table S5 Real datasets used in the experiments.

Dataset	The number of samples	The number of contigs (> 1000bp)	N50	Read length (bp)	Q20 (%)	Sequencing platform
STEC	53	255484	2642	150	96.47	Illumina MiSeq and HiSeq 2500
Water Group1	8	556732	2408	150	100	Illumina HiSeq 4000
Water Group2	5	476396	2632	150	100	Illumina HiSeq 4000
Water Group3	7	687772	2508	150	100	Illumina HiSeq 4000
MetaHIT	264	684179	3440	44, 45 or 75	71.02	Illumina Genome Analyzer II
MetaHIT (10-sample)	10	116881	4047	44 or 75	77.56	Illumina Genome Analyzer II

The term "Q20 (%)" represents the fraction of reads with an average quality > 20.

Table S6 Sample grouping information for co-assembly of the three Water Group datasets

Dataset	Runs in NCBI Sequence Read Archive database with accession number PRJNA542960
Water Group1	SRR9061275, SRR9061278, SRR9061279, SRR9061280, SRR9061281 SRR9061282, SRR9061372, SRR9061373
Water Group2	SRR9061277, SRR9061283, SRR9061370, SRR9061371, SRR9061376
Water Group3	SRR9061270, SRR9061271, SRR9061273, SRR9061274, SRR9061276 SRR9061286, SRR9061287

Table S7 Long-read sequencing datasets used for extended experiments.

Dataset	The number of samples	The number of contigs (> 1000bp)	N50	Q20 (%)	Sequencing platform
CRR344871	1	2559	313948	100	PacBio RS II
CRR344872	1	1471	924191	100	PacBio RS II
SRR10963010	1	21102	147150	99.89	PacBio Sequel
ERR9769275	1	20401	38185	100	PacBio Sequel II

The term "Q20 (%)" represents the fraction of reads with an average quality > 20.

Algorithm S1 The contrastive learning training process of COMEBin

Input: Batch size N_{bs}; the number of views V; Neural Networks f_{cov} and $f_{combine}$; features of contigs $X^{(com)}$ and $X^{(cov)}$.

Output: $f_{cov}$and$f_{combine}$

for Sampled mini-batch$\left\{\left\{x_{i,v}^{(com)}, x_{i,v}^{(cov)}\right\}_{v=1}^{V}\right\}_{i=1}^{N_{bs}}$ **do**

  for $i \in \{1, 2, \ldots, N_{bs}\}$ **do**

    for all $v \in \{1, 2, \ldots, V\}$ **do**

      $Z_{i,v}$=$f_{combine}$(concat($x_{i,v}^{(com)}$, norm(f_{cov}($x_{i,v}^{(cov)}$))))

    end for

  end for

  Update network parameters of f_{cov} and $f_{combine}$ to minimize L. L is given in Equation 12 in the main text.

end for

return f_{cov} and $f_{combine}$

2 Supplementary Note

2.1 Estimating completeness and contamination of the bins

Similar to MetaBinner [2], we utilized CheckM1 [3] to analyze one binning result and identify contigs containing single-copy genes of bacterial or archaeal domains. Subsequently, we employed the scoring strategy provided by CheckM1 [3] to estimate the contamination and completeness of each bin in all the clustering results, leveraging the obtained information.

2.2 The binning performance of COMEBin on the long-read data.

We conducted additional testing to evaluate COMEBin's performance on four long-read datasets. We included SemiBin2, SemiBin2 (long-read mode), MetaDecoder, and MetaBAT2 for comparison. Three of these datasets were previously used in SemiBin2's evaluation. Long-read assemblies were generated using flye (version 2.9.2) with the options "–pacbio-hifi" and "–meta". More details about the long-read datasets can be found in Table S7. These datasets are publicly available in the National Genomics Data Center (NGDC) under the study accession PRJCA007414 (Runs: CRR344871 and CRR344872), in the ENA under the run accession SRR10963010, and in the NCBI under the run accession ERR9769275. It's worth noting that long-read sequencing typically produces highly contiguous assemblies, resulting in fewer contigs and smaller bins (measured by the number of contigs) [4]. According to the results shown in Supplementary Fig. S10, SemiBin2 (long-read mode) performs best, followed by COMEBin.

2.3 Comparison of variants of COMEBin using different clustering methods

We conducted experiments with different variants of COMEBin, replacing the Leiden-based clustering method with InfoMap, as implemented in SemiBin1. Additionally, we employed k-means and weighted k-means for clustering, utilizing the embeddings as features, and determined bin numbers based on single-copy genes. In "weighted k-means", we assigned the weight for each contig based on its length. For Infomap, we used the same graphs converted from the embeddings as inputs, following the same methodology for automatically selecting the final result as in COMEBin. The parameters used to generate the graphs included σ in Formula 13 with values of 0.05, 0.1, 0.15, 0.2, and 0.3, along with edge ratios(proportions of edges kept for clustering) with values of 50%, 80%, and 100%. Our comparative analysis revealed that COMEBin outperforms its variants, as illustrated in Supplementary Fig. S13.

References

[1] Meyer, F., Fritz, A., Deng, Z.-L., Koslicki, D., Lesker, T.R., *et al.*: Critical assessment of metagenome interpretation: the second round of challenges. Nature Methods **19**(4), 429–440 (2022)

[2] Wang, Z., Huang, P., You, R., Sun, F., Zhu, S.: MetaBinner: a high-performance and stand-alone ensemble binning method to recover individual genomes from complex microbial communities. Genome Biology **24**(1), 1 (2023)

[3] Parks, D.H., Imelfort, M., Skennerton, C.T., Hugenholtz, P., Tyson, G.W.: CheckM: assessing the quality of microbial genomes recovered from isolates, single cells, and metagenomes. Genome Research **25**(7), 1043–1055 (2015)

[4] Pan, S., Zhao, X.M., Coelho, L.P.: SemiBin2: self-supervised contrastive learning leads to better MAGs for short- and long-read sequencing. Bioinformatics **39**(39 Suppl 1), 21–29 (2023)
