# Supplementary material for: Effective binning of metagenomic contigs using contrastive multi-view representation learning
Source: Nat Commun. 2024 Jan 17;15:585. doi: 10.1038/s41467-023-44290-z (PMC10794208; doi:10.1038/s41467-023-44290-z)
Supplement: Supplementary file 3 — Reporting Summary [file 41467_2023_44290_MOESM3_ESM.pdf]

nature portfolio

Corresponding author(s): Shanfeng Zhu

Last updated by author(s): 2023/11/29

Reporting Summary

Nature Portfolio wishes to improve the reproducibility of the work that we publish. This form provides structure for consistency and transparency in reporting. For further information on Nature Portfolio policies, see our Editorial Policies and the Editorial Policy Checklist.

Statistics

For all statistical analyses, confirm that the following items are present in the figure legend, table legend, main text, or Methods section.

n/a	Confirmed	
☐	☒	The exact sample size (*n*) for each experimental group/condition, given as a discrete number and unit of measurement
☐	☒	A statement on whether measurements were taken from distinct samples or whether the same sample was measured repeatedly
☒	☐	The statistical test(s) used AND whether they are one- or two-sided<br>*Only common tests should be described solely by name; describe more complex techniques in the Methods section.*
☒	☐	A description of all covariates tested
☒	☐	A description of any assumptions or corrections, such as tests of normality and adjustment for multiple comparisons
☐	☒	A full description of the statistical parameters including central tendency (e.g. means) or other basic estimates (e.g. regression coefficient) AND variation (e.g. standard deviation) or associated estimates of uncertainty (e.g. confidence intervals)
☒	☐	For null hypothesis testing, the test statistic (e.g. *F*, *t*, *r*) with confidence intervals, effect sizes, degrees of freedom and *P* value noted<br>*Give P values as exact values whenever suitable.*
☒	☐	For Bayesian analysis, information on the choice of priors and Markov chain Monte Carlo settings
☒	☐	For hierarchical and complex designs, identification of the appropriate level for tests and full reporting of outcomes
☒	☐	Estimates of effect sizes (e.g. Cohen's *d*, Pearson's *r*), indicating how they were calculated

Our web collection on statistics for biologists contains articles on many of the points above.

Software and code

Policy information about availability of computer code

Data collection	No software was used.
Data analysis	For the development of COMEBin, we used python v.3.7.12, pytorch v.1.10.2, hmmer v.3.1b2, scikit-learn v.0.22.1, checkm v.1.1.3, biopython v.1.76, fraggenescan v.1.31, pplacer v.1.1.alpha19, numpy v.1.19.0, leidenalg v.0.8.10, igraph v0.9.9, networkx v2.6.3, bedtools v.2.30.0, pandas v.1.3.5, hnswlib v0.6.2. The COMEBin software is freely available at https://github.com/ziyewang/COMEBin under the GNU General Public License version v3.<br><br>For generating the binning results and the analysis of the benchmarking results, we used CONCOCT 1.0.0, MaxBin2 2.2.6, MetaBAT2 2.12.1 , VAMB 4.1.3, CLMB, MetaDecoder 1.0.11, SemiBin1 1.0.0, SemiBin2 1.5.1, BWA 0.7.17, AMBER 2.0.3, CheckM2 1.0.1, GTDB-Tk v.2.3.0, MetaWRAP 1.2.1, Antismash v.6.1.1, BLASTP 2.14.1, and Resistance Gene Identifier (RGI version 6.0.2).

For manuscripts utilizing custom algorithms or software that are central to the research but not yet described in published literature, software must be made available to editors and reviewers. We strongly encourage code deposition in a community repository (e.g. GitHub). See the Nature Portfolio guidelines for submitting code & software for further information.

Data

Policy information about availability of data

All manuscripts must include a data availability statement. This statement should provide the following information, where applicable:

- Accession codes, unique identifiers, or web links for publicly available datasets
- A description of any restrictions on data availability
- For clinical datasets or third party data, please ensure that the statement adheres to our policy

There is no restriction on data availability.
All the datasets used in this study are publicly available. The simulated datasets, including CAMI mouse gut, CAMI Airways, CAMI Gastrointestinal tract, CAMI Skin, Marine GSA, Marine MA, Plant-associated GSA, Plant-associated MA, Strain-madness GSA, and Strain-madness MA, were created by CAMI II Challenge. These datasets can be accessed from the CAMI portal at https://data.cami-challenge.org. All the simulated datasets are also downloadable from their respective DOIs (CAMI mouse gut: 10.4126/FRL01-006421672; CAMI Airways, CAMI Gastrointestinal tract, and CAMI Skin: 10.4126/FRL01-006425518; Marine, Plant-associated and Strain-madness: 10.4126/FRL01-006425521). The sequence data (STEC, Water Group, and MetaHIT datasets) used in the study are publicly available in the ENA with study accessions PRJEB1775, PRJNA542960, and PRJEB2054. The sequencing reads of the BATS samples are publicly available in the NCBI with accession number PRJNA385855, and the corresponding assemblies are publicly available in the ENA with accession number PRJEB45951 For long-read datasets, the sequencing reads are publicly available in the National Genomics Data Center (NGDC) under the study accession PRJCA007414(Runs: CRR344871 and CRR344872), in the ENA under the run accession SRR10963010, and in the NCBI under the run accession ERR9769275.

Research involving human participants, their data, or biological material

Policy information about studies with human participants or human data. See also policy information about sex, gender (identity/presentation), and sexual orientation and race, ethnicity and racism.

Reporting on sex and gender	The study does not involve any human participants or human data.
Reporting on race, ethnicity, or other socially relevant groupings	The study does not involve any human participants or human data.
Population characteristics	The study does not involve any human participants or human data.
Recruitment	The study does not involve any human participants or human data.
Ethics oversight	The study does not involve any human participants or human data.

Note that full information on the approval of the study protocol must also be provided in the manuscript.

Field-specific reporting

Please select the one below that is the best fit for your research. If you are not sure, read the appropriate sections before making your selection.

☒ Life sciences ☐ Behavioural & social sciences ☐ Ecological, evolutionary & environmental sciences

For a reference copy of the document with all sections, see nature.com/documents/nr-reporting-summary-flat.pdf

Life sciences study design

All studies must disclose on these points even when the disclosure is negative.

Sample size	No calculation of sample sizes were made. We used the available public datasets (simulated datasets): CAMI Mouse gut (n=64), CAMI Skin (n=10), CAMI Airways (n=10), and CAMI Gastrointestinal tract (n=10), Marine GSA (n=10), Marine MA (n=10), Plant-associated GSA (n=21), Plant-associated MA (n=21), Strain-madness GSA (n=100) and Strain-madness MA (n=100). We also used the available public datasets (real datasets): STEC (n=53), Water group1 (n=8), Water group2 (n=5), Water group3 (n=7), METAHIT (n=264) and Bermuda-Atlantic Time-series Study (BATS) samples (n=10).
Data exclusions	No data were excluded from the analyses.
Replication	No experimental replications were performed, except for obtaining the average running time and memory usage of the binning methods on the STEC and BASTS datasets (averaged over three trials). The benchmark datasets were divided into training and holdout datasets. The method was developed based on the six training datasets and tested on the ten holdout datasets. Additionally, validation was conducted using samples from the Bermuda-Atlantic Time-series Study (BATS).
Randomization	To further demonstrate the usability of COMEBin across different binning modes, we randomly selected ten samples from the MetaHIT sequencing samples (n=264) and evaluated COMEBin's performance in single-sample, co-assembly, and multi-sample modes. The allocation was random, and the accession codes for the samples are given in the supplementary file (Table S2).
Blinding	Investigators were not blinded to the datasets; however, the COMEBin development utilized the training datasets, while other datasets were held out for validation.

Reporting for specific materials, systems and methods

We require information from authors about some types of materials, experimental systems and methods used in many studies. Here, indicate whether each material, system or method listed is relevant to your study. If you are not sure if a list item applies to your research, read the appropriate section before selecting a response.

Materials & experimental systems

n/a	Involved in the study	
☒	☐	Antibodies
☒	☐	Eukaryotic cell lines
☒	☐	Palaeontology and archaeology
☒	☐	Animals and other organisms
☒	☐	Clinical data
☒	☐	Dual use research of concern
☒	☐	Plants

Methods

n/a	Involved in the study	
☒	☐	ChIP-seq
☒	☐	Flow cytometry
☒	☐	MRI-based neuroimaging
